# Characterization and Expression Analysis of the C-Type Lectin Ladderlectin in *Litopenaeus vannamei* Post-WSSV Infection

**DOI:** 10.3390/biology13100758

**Published:** 2024-09-24

**Authors:** Qian Xue, Bingbing Yang, Kun Luo, Sheng Luan, Jie Kong, Qiang Fu, Jiawang Cao, Baolong Chen, Ping Dai, Qun Xing, Xupeng Li, Xianhong Meng

**Affiliations:** 1 State Key Laboratory of Mariculture Biobreeding and Sustainable Goods, Yellow Sea Fisheries Research Institute, Chinese Academy of Fishery Sciences, Qingdao 266071, China; 13011754639@163.com (Q.X.); nmzl19951209@163.com (B.Y.); luokun@ysfri.ac.cn (K.L.); luansheng@ysfri.ac.cn (S.L.); kongjie@ysfri.ac.cn (J.K.); oucfuq@163.com (Q.F.); weifengchui555@yeah.net (J.C.); 1320040091@163.com (B.C.); daiping54@163.com (P.D.); 2 Laboratory for Marine Fisheries Science and Food Production Processes, Qingdao Marine Science and Technology Center, Qingdao 266237, China; 3 BLUP Aquabreed Co., Ltd., Weifang 261311, China; xingqun527@163.com

**Keywords:** *Litopenaeus vannamei*, C-type lectin, white spot syndrome virus, ladderlectin, RNAi, innate immunity

## Abstract

**Simple Summary:**

The outbreak of white spot syndrome poses a significant threat to the shrimp farming industry. To explore the interaction between *Litopenaeus vannamei* and the pathogenic white spot syndrome virus (WSSV), this study cloned and analyzed an important pattern recognition receptor, the C-type lectin ladderlectin (*LvLL*). The results revealed that *LvLL* in *L*. *vannamei* promoted WSSV replication, thereby affecting shrimp survival rates. This study demonstrated the role of *LvLL* in the resistance of *L*. *vannamei* to WSSV, providing theoretical insights into the mechanisms of shrimp resistance to the virus.

**Abstract:**

C-type lectins are known for agglutination activity and play crucial roles in regulating the prophenoloxidase (proPO) activation system, enhancing phagocytosis and encapsulation, synthesizing antimicrobial peptides, and mediating antiviral immune responses. This work cloned a C-type lectin, ladderlectin (*LvLL*), from *Litopenaeus vannamei*. *LvLL* comprised a 531 bp open reading frame (ORF) that encoded 176 amino acids. The predicted LvLL protein included a signal peptide and a CLECT domain. LvLL was predicted to feature a transmembrane region, suggesting it may be a transmembrane protein. *LvLL* was predominantly expressed in the shrimp’s hepatopancreas. After WSSV infection, *LvLL* expression in the hepatopancreas increased significantly by 11.35-fold after 228 h, indicating a general upregulation. Knockdown of *LvLL* resulted in a significant decrease in WSSV viral load and a notable increase in shrimp survival rates. Additionally, knockdown of *LvLL* led to a significant downregulation of apoptosis-related genes *Bcl-2* and caspase 8 and a significant upregulation of *p53* and *proPO* in WSSV-infected shrimp. This study showed that *LvLL* played a vital role in the interaction between *L*. *vannamei* and WSSV.

## 1. Introduction

*Litopenaeus vannamei* is a globally significant cultivated shrimp species known for its nutritional value, rapid growth, disease resistance, and high economic yield. As aquaculture expands, frequent disease outbreaks lead to severe economic losses, with viral diseases posing a substantial threat to global shrimp farming [1,2]. White spot syndrome virus (WSSV) is considered one of the most economically devastating pathogens in shrimp aquaculture worldwide. WSSV, an oval-shaped double-stranded DNA virus measuring 250–380 nm in length and 80–120 nm in diameter, belongs to the *Whispovirus* genus of the *Nimaviridae* family [3]. WSSV has a broad host range and can cause a 100% mortality rate in shrimp [4,5]. Therefore, studying the interaction mechanism between shrimp and WSSV is crucial for advancing shrimp aquaculture.

*L*. *vannamei* primarily relies on innate immunity to recognize and defend against pathogens [6]. During bacterial or viral infections, the shrimp’s innate immune system activates various pattern recognition receptors (PRRs) to identify pathogen-associated molecular patterns (PAMPs). This activation triggers signal transduction pathways, leading to the synthesis of antimicrobial peptides, activation of the proPO system, and modulation of apoptosis-related gene expression. These processes work together to control and eliminate pathogens [7,8]. The accurate identification of invasive infections is crucial for initiating the innate immune response. C-type lectins (CTLs), a key class of PRRs, play an essential role in this process. These CTLs typically contain at least one carbohydrate recognition domain (CRD), consisting of approximately 110–130 amino acids [9]. The CRD forms a characteristic double-loop structure stabilized by two disulfide bonds and usually includes calcium-binding sites [10,11]. Through their CRDs, CTLs specifically recognize highly conserved PAMPs on pathogen surfaces, thereby triggering a cascade of immune responses [12,13].

Besides acting as PRRs to recognize PAMPs, CTLs play crucial roles in various immune responses, including antibacterial and antiviral activities, the regulation of the proPO activation system, the mediation of cell–cell interactions, and the promotion of phagocytosis, encapsulation, and melanization [14,15,16]. In *L*. *vannamei*, LvCTLD recruits hemocytes and stimulates the proPO activation system at virus infection sites to exert immune defense [17]. In *Portunus trituberculatus*, PtCLec1 interacts with a variety of pathogenic microorganisms and exhibits agglutination and antibacterial activities [18]. FmLdlr contributes to the shrimp’s immune defense mainly through the action of its CRD, which is capable of bacterial agglutination, enhances phagocytosis and encapsulation, has antimicrobial activity, and binds to WSSV proteins [19].

Upon WSSV invasion in crustaceans, CTLs can function as viral binding proteins or receptors, either inhibiting or promoting WSSV infection. In *L*. *vannamei*, LvLec1 acts as a PRR in the innate immune system, playing a defensive role against both WSSV and bacterial infections [20]. It was reported that LvCTL1 is a mannose-binding CTL that binds to the envelope proteins of WSSV to exert its antiviral activity [21]. Only a few CTLs have been found to promote WSSV infection in crustaceans. For example, the transmembrane CTL PcTlec in *Procambarus clarkii* may act as a WSSV receptor by interacting with VP28, thereby facilitating WSSV infection [22]. Similarly, LvCTL 4.2 in *L*. *vannamei* and MjsvCL in *Marsupenaeus japonicus* interact with WSSV proteins, promoting infection [23,24].

By analyzing transcriptome sequencing data (NCBI: PRJNA1101112) from our team’s previous research on resistance to WSSV, the significant gene ladderlectin (*LvLL*) was identified as the focus of this study. The complete ORF sequence of *LvLL* was obtained using PCR and Sanger sequencing, followed by bioinformatics analysis. To validate *LvLL*’s role in the innate immune response against WSSV, mRNA expression profiles were analyzed in the hepatopancreas, gill, muscle, and eyestalk of both healthy and WSSV-infected *L*. *vannamei*. Additionally, RNA interference (RNAi) was used to knock down *LvLL* expression, and the function of *LvLL* in the immune response of *L*. *vannamei* against WSSV was further analyzed. The regulation of apoptosis and the proPO activation system in shrimp after *LvLL* knockdown was also investigated. Furthermore, single nucleotide polymorphism (SNP) sites within the gene were identified and analyzed for their association with resistance to WSSV.

## 2. Materials and Methods

### 2.1. Experimental Animals

All *L*. *vannamei* were provided by BLUP Aquabreed Co., Ltd. (Weifang, China). The shrimp were housed in the experimental platform (1 m^2^ × 1.2 m) at the Yellow Sea Fisheries Research Institute, Chinese Academy of Fishery Sciences (Qingdao, China). Daily management followed a standardized protocol. Before the experiment, the shrimp were acclimated for three days in a stable rearing environment, with water changes performed daily (half the water volume each time) while maintaining water salinity at 29 ± 1 and temperature at 26 ± 1 °C, with adequate dissolved oxygen supplied by aerators. Feeding was carried out three times daily. For cloning the *LvLL* sequence and analyzing *LvLL* expression after WSSV infection, shrimp weighing 2.5–3.5 g and measuring 6.0–6.6 cm in length were used. For the RNAi experiment, shrimp weighing 2.7–3.3 g and measuring 5.5–6.3 cm in length were used.

### 2.2. Tissue Distribution and Expression Profiles of LvLL after WSSV Challenge

Different tissues, including the hepatopancreas, gill, muscle, and eyestalk, were sampled from five healthy *L*. *vannamei* to analyze the *LvLL* tissue distribution. In the WSSV challenge experiment, 150 healthy *L*. *vannamei* were randomly divided into three groups. The shrimp were fed approximately 10 mg of shrimp muscle tissue stained with red food dye containing 1 × 10^6^ copies/mg of WSSV. At 24, 48, 72, 96, 144, 192, and 228 h after WSSV infection, five individuals were randomly selected to collect the hepatopancreas, gill, muscle, and eyestalk, respectively. All samples were stored in RNAstore reagent (Beijing Solarbio Science & Technology Co., Ltd., Beijing, China) at −80 °C.

### 2.3. RNA Extraction and cDNA Synthesis

Total RNA was extracted from the hepatopancreas, gill, muscle, and eyestalk of both healthy and WSSV-infected shrimp using the RNA-Easy isolation reagent (Vazyme Biotech Co., Ltd., Nanjing, China). The RNA quality was assessed using a NanoPhotometer^®^ spectrophotometer (IMPLEN, Munich, Germany) and 1% agarose gel electrophoresis. The extracted RNA was then reverse-transcribed into cDNA using the HiScript^®^ III RT SuperMix for qPCR (+gDNA wiper) kit (Vazyme Biotech Co., Ltd., Nanjing, China) for subsequent qRT-PCR validation.

### 2.4. Molecular Cloning and Sequencing of LvLL

On the basis of the *LvLL* reference sequence from the NCBI database (XM_027364192.1), primers (LvLL-F and LvLL-R) (Table 1) were designed using Primer 3 online (https://primer3.ut.ee/) to amplify the *LvLL* ORF sequence. The cDNA synthesized from total RNA extracted from the hepatopancreas of *L*. *vannamei* was used as the template. Quick Taq HS DyeMix (TOYOBO, Shanghai, China) was used for PCR amplification of the *LvLL* ORF sequence. The 50 μL reaction mixture consisted of 25 μL 2× Quick Taq HS DyeMix, 1 μL of each primer (10 μM), 4 μL of cDNA solution (100 ng/μL), and 19 μL of double-distilled water. The PCR program included an initial denaturation at 94 °C for 2 min, followed by 35 cycles of 94 °C for 30 s, 55 °C for 30 s, and 68 °C for 1 min, with a final extension at 68 °C for 5 min. The size of the PCR product was verified by 1% agarose gel electrophoresis, followed by Sanger sequencing. The obtained sequence data were compared with the NCBI database reference sequence to accurately verify the ORF sequence for subsequent bioinformatics analysis.

### 2.5. Bioinformatics Analysis

Using EditSeq 7.1 software (https://www.dnastar.com/, accessed on 20 August 2023), the ORF sequence of *LvLL* was translated into an amino acid sequence. The ExPASy-ProtParam tool (https://web.expasy.org/protparam/, accessed on 20 August 2023) was employed to predict the molecular weight (MW), theoretical isoelectric point (pI), and instability index of the protein. The NetNGlyc 1.0 Server (http://www.cbs.dtu.dk/services/NetNGlyc/, accessed on 20 August 2023) was employed to predict glycosylation sites. Transmembrane regions of the protein were investigated with TMHMM (http://www.cbs.dtu.dk/services/TMHMM/, accessed on 20 August 2023). Signal peptide structures were predicted using SignalP 4.1 software (https://services.healthtech.dtu.dk/services/SignalP-4.1/, accessed on 20 August 2023). The NetPhos 3.1 Server (https://services.healthtech.dtu.dk/services/NetPhos-3.1/, accessed on 20 August 2023) was used to predict phosphorylation sites. The SMART online tool (https://smart.embl.de/, accessed on 20 August 2023) was used to predict protein functional domains. The secondary structure of the protein was predicted using SOPMA (https://npsa-prabi.ibcp.fr/, accessed on 20 August 2023). DNAMAN 6.0 (Lynnon Biosoft, San Ramon, CA, USA, accessed on 20 August 2023) was used for homology sequence alignment with other species. MEGA 7.0 software (https://www.megasoftware.net/, accessed on 20 August 2023) was utilized to construct a phylogenetic tree of ladderlectin using the neighbor-joining method (NJ).

### 2.6. RT-PCR and Statistical Analysis

SYBR Green Real-time PCR Master Mix (TOYOBO, Shanghai, China) was used to perform the RT-PCR assay on an Applied Biosystems^TM^ QuantStudio 1 Real-Time PCR quantifier (Applied Biosystems, Foster City, CA, USA). The cDNA solution, synthesized by reverse transcription of the extracted total RNA, served as the reaction template. The primer sequences (qLvLL-F, qLvLL-R, 18S-F, and 18S-R) are listed in Table 1. The 18S ribosomal RNA (18S rRNA) was used as an internal reference gene. The 20 μL reaction mixture contained 10 μL of SYBR Green Real-time PCR Master Mix, 0.8 μL of each primer (10 μM), 2 μL of cDNA solution (100 ng/μL), and 6.4 μL of double-distilled water. The reaction program included 95 °C for 60 s, followed by 40 cycles of 95 °C for 15 s, 60 °C for 15 s, and 72 °C for 45 s. Each sample was tested in triplicate. The 2^−ΔΔCt^ method was used to analyze *LvLL* expression levels in different tissues after WSSV infection. Additionally, IBM SPSS Statistics 25.0 software (https://www.ibm.com/spss/, accessed on 12 September 2023) was used to conduct independent sample *t*-tests on the experimental data, with *p* < 0.05 indicating significant differences and *p* < 0.01 indicating highly significant differences.

### 2.7. Synthesis of Double-Stranded RNA

Double-stranded RNA (dsRNA) targeting the *LvLL* (termed *dsLvLL*) was synthesized via in vitro transcription, with dsRNA targeting GFP (termed *dsGFP*) serving as control. The cDNA synthesized from the total RNA extracted from the hepatopancreas of *L*. *vannamei* was used as the template. PCR with T7 promoter-specific primers (dsLvLL-Fi and dsLvLL-Ri) (Table 1) was used to generate the DNA template for *dsLvLL* synthesis. A pET28a plasmid containing the GFP sequence was used as the template for *dsGFP* synthesis with specific primers (dsGFP-Fi and dsGFP-Ri) (Table 1). The purified PCR products served as templates for dsRNA synthesis using the In Vitro Transcription T7 Kit (for siRNA Synthesis) (Takara, Dalian, China). The reaction was performed in a 20 μL volume containing 2 μL of 10× Transcription Buffer, 2 μL of ATP Solution, 2 μL of GTP Solution, 2 μL of CTP Solution, 2 μL of UTP Solution, 0.5 μL of RNase Inhibitor, 2 μL of T7 RNA Polymerase, 3 μL of DNA templates (1 μg), and 4.5 μL of double-distilled water. The mixture was incubated at 42 °C for 2 h, followed by the addition of 2 μL of Rnase-free Dnase I and further incubation at 37 °C for 30 min. The synthesized dsRNA was stored at −80 °C.

### 2.8. Analysis of dsRNA Interference Efficiency

To assess RNAi efficiency, 60 healthy shrimp were randomly divided into two groups: *dsGFP* + WSSV and *dsLvLL* + WSSV. The *dsGFP* + WSSV group received an injection of 10 μL of *dsGFP* (3 μg/μL) into the third abdominal segment of the shrimp as a control, while the *dsLvLL* + WSSV group received an injection of 10 μL of *dsLvLL* (3 μg/μL). A second injection of 10 μL of dsRNA was administered 24 h later to enhance RNAi efficiency, followed by an injection of 20 μL of WSSV viral suspension (4.7 × 10^6^ copies). Hepatopancreas samples were collected at 24 and 48 h after WSSV injection, stored in liquid nitrogen, and transferred to a −80 °C freezer for future RNA extraction and RT-PCR analysis to determine *LvLL* expression levels.

### 2.9. Survival Rate Analysis

A total of 120 healthy shrimp were randomly assigned to four groups: PBS, *dsGFP* + WSSV, WSSV, and *dsLvLL* + WSSV. Each group was injected with 10 μL of 1 × PBS, *dsGFP* (3 μg/μL), an empty syringe, or *dsLvLL* (3 μg/μL) into the third abdominal segment, respectively. After 24 h, the PBS group received another 10 μL of 1 × PBS, while the other groups received another 10 μL injection of their respective dsRNA. This was followed by an injection of 20 μL of WSSV viral suspension (4.7 × 10^6^ copies) in all groups except the PBS group. Survival rates were measured at different time points (0, 12, 24, 36, 48, and 72 h) after WSSV injection. The survival rates were statistically analyzed using the log-rank test with GraphPad Prism 8.0 (https://www.graphpad.com/, accessed on 16 October 2023) to determine significant differences between groups.

### 2.10. Investigating Immune Response and Gene Expression after LvLL Knockdown

#### 2.10.1. Experimental Setting

A total of 120 healthy shrimp were randomly assigned to four groups: PBS, *dsGFP* + WSSV, WSSV, and *dsLvLL* + WSSV. Each group was injected with 10 μL 1 × PBS, *dsGFP* (3 μg/μL), an empty syringe, or *dsLvLL* (3 μg/μL) into the third abdominal segment, respectively. After 24 h, the PBS group was injected with another 10 μL of 1 × PBS, while the other groups received another 10 μL injection of their respective dsRNA, followed by 20 μL of WSSV viral suspension (4.7 × 10^6^ copies) in all groups except the PBS group. The hepatopancreas and muscle of the shrimp were collected at 0, 36, and 48 h after WSSV infection and stored at −80 °C. The experiment was conducted in triplicate.

#### 2.10.2. TaqMan RT-PCR

Genomic DNA was extracted from the muscle of *L*. *vannamei* 48 h after WSSV infection following the knockdown *LvLL*. The extraction was performed using the TIANamp Marine Animals DNA Kit (Tiangen Biotech Co., Ltd., Beijing, China). The quality of the extracted DNA was then assessed. The WSSV viral load was quantified using TaqMan RT-PCR with specific primers (WSSV-F and WSSV-R) and a probe (WSSV probe), as listed in Table 1. The Applied Biosystems^TM^ QuantStudio 1 Real-Time PCR Quantifier (Applied Biosystems, Foster City, CA, USA) and THUNDERBIRD^TM^ Probe qPCR Mix (TOYOBO, Shanghai, China) were used. The 20 μL reaction mixture contained 10 μL of THUNDERBIRD Probe qPCR Mix, 0.6 μL of each primer (10 μM), 0.4 μL of WSSV probe (10 μmol/L), 0.1 μL of ROX reference dye, 2 μL of DNA solution, and 6.3 μL of double-distilled water. The reaction program included 95 °C for 30 s, followed by 40 cycles of 95 °C for 5 s and 60 °C for 34 s. The experiment was performed in triplicate. The concentration of WSSV DNA was determined to assess the viral load.

#### 2.10.3. Gene Expression Analysis

To further investigate the function of *LvLL* in the innate immune response to WSSV in *L*. *vannamei*, RT-PCR was used to measure the expression levels of innate immune-related genes, including B-cell lymphoma-2 (*Bcl-2*) (XM_027353493.1), caspase 8 (XM_027383230.1), p53-like (*p53*) (XM_027365892.1), and *proPO* (AY723296). Total RNA was extracted from the hepatopancreas of the *dsGFP* + WSSV and *dsLvLL* + WSSV groups at 0, 36, and 48 h after WSSV infection and then reverse-transcribed into cDNA. The specific primers used for RT-PCR (Bcl-2-F, Bcl-2-R, caspase 8-F, caspase 8-R, p53-F, p53-R, proPO-F, proPO-R, 18S-F, and 18S-R) are listed in Table 1.

### 2.11. Screening of LvLL SNP and Correlation Analysis with WSSV Resistance

A total of 1152 *L*. *vannamei* from 32 families were individually and quantitatively fed with WSSV bait (using the method in Section 2.2) to assess WSSV resistance. The experiment concluded after 530 h, with 949 shrimp deaths and 203 survivors. The average survival time for deceased shrimp was (281 ± 145.48) h, and the muscle tissues were stored at −80 °C. Forty early-deceased shrimp (survival time 140.17 ± 17.06 h) were selected as the susceptible group (S group), while forty late-surviving shrimp were designated as the resistant group (R group) for SNP screening and WSSV resistance analysis. The RNA extraction and cDNA synthesis methods were the same as those described above. Specific primers (LvLL-F and LvLL-R) (Table 1) were used to amplify the ORF sequence of *LvLL*, followed by Sanger sequencing. SeqMan software (https://www.dnastar.com/, accessed on 8 December 2023) was used to align the *LvLL* ORF sequences with the NCBI reference sequence, identifying all SNPs from overlapping peaks in the Sanger chromatograms.

SNP genotype data were used to calculate genotype frequencies and genetic diversity indices, including the observed heterozygosity (*H*_o_), expected heterozygosity (*H*_e_), minor allele frequency (MAF), polymorphic information content (PIC), and Hardy–Weinberg equilibrium (HWE), using PLINK 1.9 software [25]. PIC values below 0.25 indicated low polymorphism, values between 0.25 and 0.50 indicated moderate polymorphism, and values above 0.50 indicated high polymorphism. Chi-square tests (χ^2^) using IBM SPSS Statistics 25.0 software (https://www.ibm.com/spss/, accessed on 8 December 2023) were used to determine significant (*p* < 0.05) and highly significant (*p* < 0.01) differences, correlating SNPs with WSSV resistance traits in *L*. *vannamei*.

## 3. Results

### 3.1. Characterization and Analysis of LvLL

**Figure 1:**
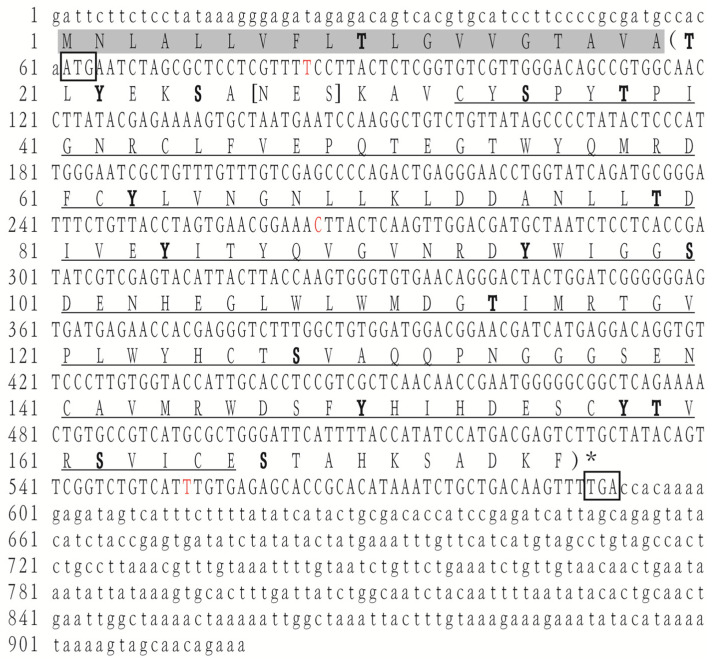
The cDNA sequence and deduced amino acid sequence of the *LvLL* gene. The start and stop codons are indicated by boxes. The predicted CLECT structural domain is underlined. The predicted transmembrane regions are indicated by parentheses. The predicted phosphorylation sites are indicated in bold font. SNP sites are marked in red font.

The ORF of *LvLL* was 531 bp, encoding 176 amino acids (Figure 1). It was predicted to contain 20 negatively charged amino acid residues (Asp + Glu) and 11 positively charged residues (Arg + Lys). The MW was 19.89 kDa, and the pI was 4.84. The instability index was 37.41, classifying it as a stable protein. The aliphatic index was 81.36, and the average hydropathicity was −0.124. The transmembrane region was predicted to span amino acids 20–176, with a signal peptide structure located at positions 1–19. Glycosylation site analysis predicted an N-glycosylation site with an NES motif at position 27. Phosphorylation site analysis showed a total of 18 phosphorylation sites, with 6 each for serine, threonine, and tyrosine. A CLECT domain was present at 33–166 (Figure 1). Secondary structure prediction, excluding the signal peptide, revealed that the protein mainly consists of random coils (53.41%), α-helices (27.84%), and extended strands (18.75%).

### 3.2. Homologous and Phylogenetic Analysis of Ladderlectin

**Figure 2:**
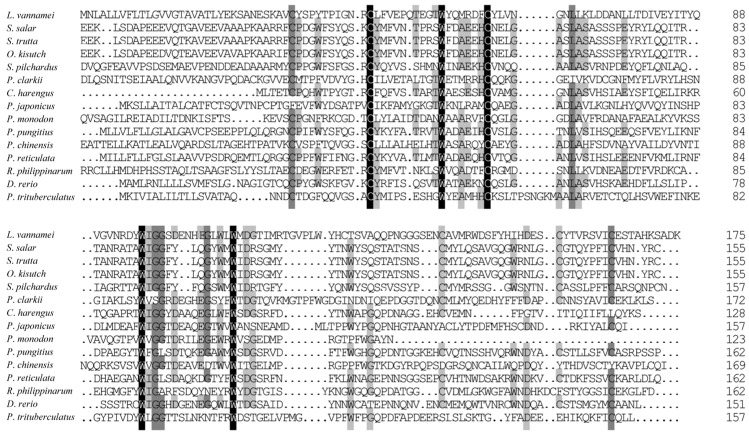
Multiple sequence alignment of ladderlectin amino acid sequences. The 100% identical residues are indicated by black shading, 75% identical residues are indicated by dark gray shading, and 50% identical residues are indicated by light gray shading. The GenBank accession numbers of ladderlectin amino acid sequences are as follows: *L*. *vannamei* (XP_027219993.1), *S*. *salar* (XP_045578907.1), *S*. *trutta* (XP_029622341.1), *O*. *kisutch* (XP_031670965.1), *S*. *pilchardus* (XP_062407595.1), *P*. *clarkii* (XP_045588190.1), *Clupea harengus* (XP_042560807.1), *P*. *japonicus* (XP_042881645.1), *P*. *monodon* (XP_037799159.1), *Pungitius pungitius* (XP_037330723.2), *P*. *chinensis* (XP_047499869.1), *Poecilia reticulata* (XP_008419991.1), *R. philippinarum* (XP_060552084.1), *D*. *rerio* (XP_001337601.1), *P*. *trituberculatus* (XP_045115152.1).

BLAST analysis revealed that the deduced amino acid sequence of ladderlectin from *L*. *vannamei* (XP_027219993.1) shared moderate similarity with other species (Figure 2), such as 42.86% similarity with *Salmo salar* (XP_045578907.1), *Salmo trutta* (XP_029622341.1), and *Oncorhynchus kisutch* (XP_031670965.1). The homology percentages with other species were as follows: *Sardina pilchardus* (XP_062407595.1) at 35.94%, *P*. *clarkii* (XP_045588190.1) at 33.82%, *Clupea harengus* (XP_042560807.1) at 33.33%, *Penaeus japonicus* (XP_042881645.1) at 31.36%, *Penaeus monodon* (XP_037799159.1) at 31.00%, *Pungitius pungitius* (XP_037330723.2) at 29.66%, *Penaeus chinensis* (XP_047499869.1) at 29.45%, *Poecilia reticulata* (XP_008419991.1) at 29.06%, *Ruditapes philippinarum* (XP_060552084.1) at 25.90%, *Danio rerio* (XP_001337601.1) at 25.17%, and *P*. *trituberculatus* (XP_045115152.1) at 22.54%.

**Figure 3:**
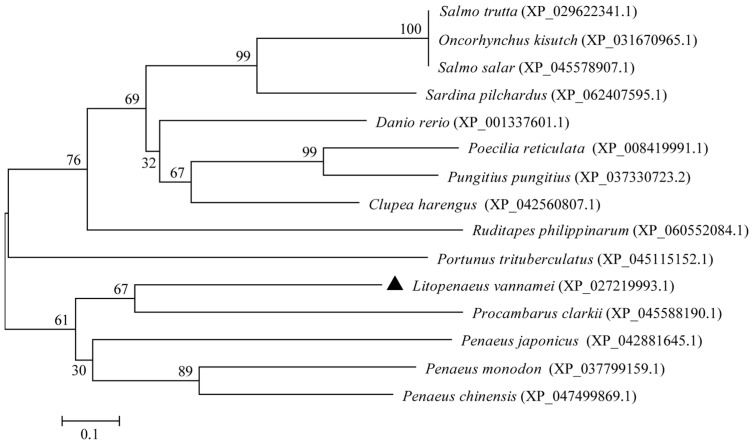
Phylogenetic tree analysis of ladderlectin. The LvLL marker of *L*. *vannamei* is “▲”.

The phylogenetic tree was constructed on the basis of the multiple sequence alignment of selected vertebrates and invertebrates (Figure 3). The tree topology was divided into two distinct clades. The results of the phylogenetic analysis were consistent with the taxonomic positions of the species. Ladderlectin from *L*. *vannamei*, *P*. *clarkii*, *P*. *japonicus*, *P*. *monodon*, and *P*. *chinensis* clustered together in a main clade.

### 3.3. Expression Profiles of LvLL after WSSV Infection

**Figure 4:**
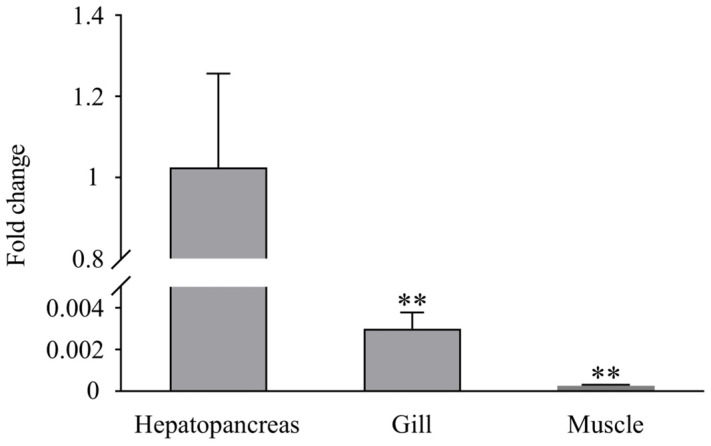
Expression profiles of *LvLL* in hepatopancreas, gill, and muscle of healthy *L*. *vannamei*. (**: *p* < 0.01).

RT-PCR analysis showed that *LvLL* was expressed in the hepatopancreas, gill, and muscle of healthy *L*. *vannamei*, with the highest expression observed in the hepatopancreas. The expression of *LvLL* in the gill was approximately 0.003-fold (*p* < 0.01) that in the hepatopancreas, while in the muscle, it was about 0.00026-fold (*p* < 0.01) that of the hepatopancreas. No *LvLL* expression was detected in the eyestalk of the shrimp (Figure 4).

**Figure 5:**
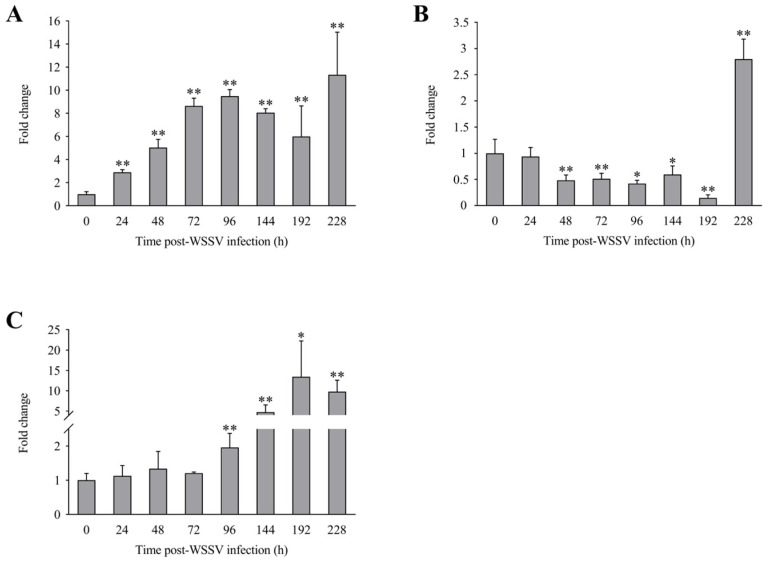
Expression profiles of *LvLL* in hepatopancreas, gill, and muscle of *L*. *vannamei* after WSSV infection. (**A**): Hepatopancreas, (**B**): gill, (**C**): muscle (*: *p* < 0.05, **: *p* < 0.01).

The expression levels of *LvLL* in the hepatopancreas of *L*. *vannamei* at 24, 48, 72, 96, 144, 192, and 228 h after WSSV infection were approximately 2.89 ± 0.23 (*p* < 0.01), 5.05 ± 0.70 (*p* < 0.01), 8.64 ± 0.67 (*p* < 0.01), 9.48 ± 0.58 (*p* < 0.01), 8.06 ± 0.35 (*p* < 0.01), 5.99 ± 2.66 (*p* < 0.01), and 11.35 ± 3.69 (*p* < 0.01) fold that of the control group, respectively (Figure 5A). In the gill, the *LvLL* expression levels at the same time points were approximately 0.93 ± 0.17, 0.48 ± 0.10 (*p* < 0.01), 0.51 ± 0.11 (*p* < 0.01), 0.42 ± 0.06 (*p* < 0.05), 0.59 ± 0.16 (*p* < 0.05), 0.15 ± 0.06 (*p* < 0.01), and 2.80 ± 0.38 (*p* < 0.01) fold that of the control group, respectively (Figure 5B). In the muscle, the *LvLL* expression levels at these times were approximately 1.13 ± 0.30, 1.34 ± 0.50, 1.21 ± 0.03, 1.96 ± 0.41 (*p* < 0.01), 4.72 ± 1.78 (*p* < 0.01), 13.43 ± 8.81 (*p* < 0.05), and 9.78 ± 2.82 (*p* < 0.01) fold that of the control group, respectively (Figure 5C).

### 3.4. WSSV Infection Was Suppressed after LvLL Knockdown

**Figure 6:**
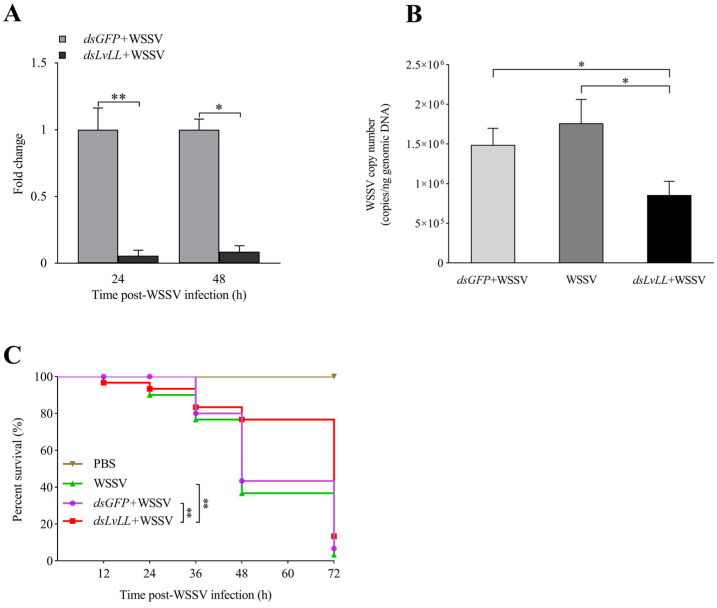
WSSV infection was suppressed after knocking down *LvLL*. (**A**): The knockdown efficiency of *LvLL* in the hepatopancreas at 24 and 48 h after WSSV infection. The knockdown efficiencies were 94.00% and 91.00% at 24 and 48 h after WSSV infection, respectively. (**B**): The WSSV viral load in the *dsGFP* + WSSV, WSSV, and *dsLvLL* + WSSV groups after *LvLL* knockdown. (**C**): The survival rate of *L*. *vannamei* after WSSV infection. *: *p* < 0.05, **: *p* < 0.01.

The RNAi efficiency results showed that the knockdown efficiencies were 94.00% and 91.00% at 24 and 48 h after WSSV infection, respectively, and the expression levels of *LvLL* in the hepatopancreas of the *dsLvLL* + WSSV group were approximately 0.06 ± 0.04 (*p* < 0.01) and 0.09 ± 0.05 (*p* < 0.05) fold higher that of the control group (*dsGFP* + WSSV) (Figure 6A), indicating successful knockdown of *LvLL* in shrimp.

At 48 h after WSSV infection, the viral loads of WSSV in the *dsGFP* + WSSV group of the shrimp were 1.49 × 10^6^ ± 2.10 × 10^5^ copies/ng; in the WSSV group, they were 1.76 × 10^6^ ± 3.01 × 10^5^ copies/ng; and in the *dsLvLL* + WSSV group, they were 8.55 × 10^5^ ± 1.76 × 10^5^ copies/ng. The WSSV viral load in the *dsLvLL* + WSSV group of shrimp was significantly lower than that in the *dsGFP* + WSSV group (*p* < 0.05) and the WSSV group (*p* < 0.05) (Figure 6B).

The survival rate showed that there was no mortality in the PBS group, with a survival rate of 100%. There was no significant difference in survival rates between the *dsGFP* + WSSV and the WSSV group. After 72 h of WSSV infection, the survival rates for the WSSV, *dsGFP* + WSSV, *dsLvLL* + WSSV, and PBS groups were 3.33%, 6.67%, 13.33%, and 100.00%, respectively. The survival rate of the *dsLvLL* + WSSV group was significantly higher than that of the *dsGFP* + WSSV and WSSV groups (*p* < 0.01) after WSSV infection (Figure 6C).

### 3.5. Expression Profiles of Genes Related to Innate Immunity after LvLL Knockdown

**Figure 7:**
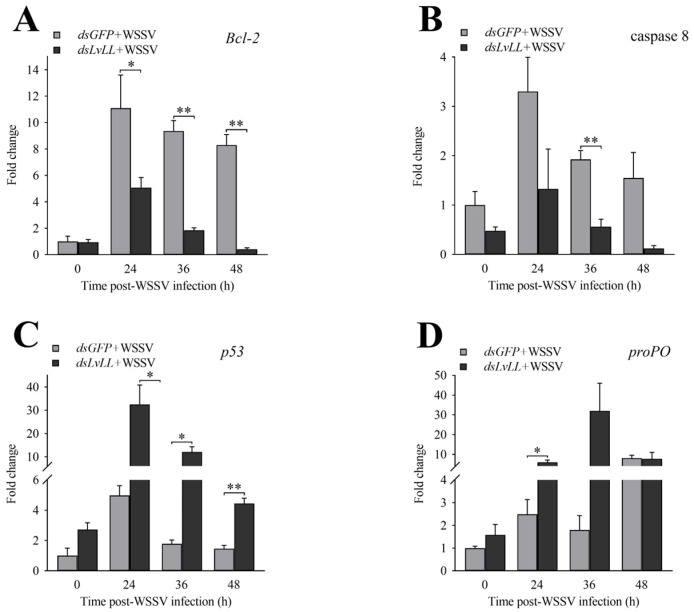
Expression profiles of immune-related genes after *LvLL* knockdown. (**A**): The expression levels of *Bcl-2* at different post-WSSV infection time points after *LvLL* knockdown. (**B**): The expression levels of caspase 8 at different post-WSSV infection time points after *LvLL* knockdown. (**C**): The expression levels of *p53* at different post-WSSV infection time points after *LvLL* knockdown. (**D**): The expression levels of *proPO* at different post-WSSV infection time points after *LvLL* knockdown. *: *p* < 0.05, **: *p* < 0.01.

After *LvLL* knockdown, the expression levels of *Bcl-2* in the *dsLvLL* + WSSV group were approximately 0.46-fold (*p* < 0.05), 0.20-fold (*p* < 0.01), and 0.05-fold (*p* < 0.01) that of the dsGFP + WSSV group at 24, 36, and 48 h after WSSV infection, respectively (Figure 7A). The expression levels of caspase 8 were approximately 0.40-, 0.29- (*p* < 0.01), and 0.08-fold that of the *dsGFP* + WSSV group at the same time points (Figure 7B). The expression levels of *p53* were approximately 6.54-fold (*p* < 0.05), 6.80-fold (*p* < 0.05), and 3.05-fold (*p* < 0.01) that of the *dsGFP* + WSSV group at the same time points (Figure 7C). The expression levels of *proPO* were approximately 2.41- (*p* < 0.05), 17.78-, and 0.97-fold that of the *dsGFP* + WSSV group at the same time points (Figure 7D).

### 3.6. SNP Detection and Polymorphism Analysis

Sequencing results identified three SNPs in the ORF of *LvLL* in *L*. *vannamei*, located at 23, 204, and 492 bp. These sites were designated as SNP1, SNP2, and SNP3, respectively (Table 2). All three were synonymous mutations: SNP1 (T/C) encoded phenylalanine (F), SNP2 (C/T) encoded asparagine (N), and SNP3 (T/A) encoded isoleucine (I).

Polymorphism analysis of *LvLL* SNPs revealed that in the S group, *H*_o_ ranged from 0.025 to 0.450, *H*_e_ ranged from 0.025 to 0.455, and MAF ranged from 0.013 to 0.350. PIC ranged from 0.024 to 0.351, with SNP3 exhibiting low polymorphism, while SNP1 and SNP2 exhibited moderate polymorphism. SNP3 had an MAF value below 0.05. The HWE results indicated that SNP1 deviated from the HWE, while SNP2 and SNP3 conformed to the HWE in the S group (Table 2). In the R group, *H*_o_ ranged from 0.000 to 0.600, *H*_e_ ranged from 0.000 to 0.495, MAF ranged from 0.000 to 0.450, and PIC ranged from 0.000 to 0.372. SNP3 showed low polymorphism, while SNP1 and SNP2 showed moderate polymorphism. SNP1 had an MAF value below 0.05. The HWE results indicated that SNP1 deviated from the HWE, while SNP2 and SNP3 conformed to the HWE in the R group (Table 2).

### 3.7. SNPs Associated with WSSV Resistance

χ^2^ analysis was used to assess the association between the three SNPs in the *LvLL* ORF and WSSV resistance traits. The χ^2^ values for SNP1, SNP2, and SNP3 were 5.230, 2.763, and 1.013, respectively. None of the SNPs showed a significant association with WSSV resistance traits (Table 3).

## 4. Discussion

CTLs are an important superfamily of immune proteins found in all metazoans. They typically contain one or more CRDs, which play a crucial role in recognizing and binding carbohydrates on the surfaces of pathogens. CTLs bind to glycosyl molecules via EPN and QPD motifs, recognizing mannose and galactose, respectively [26]. This study identified a transmembrane CTL, *LvLL*, in *L*. *vannamei*. The ORF of *LvLL* is 531 bp, encoding 176 amino acids, and it includes a signal peptide, a transmembrane region, and a CRD. CRDs recognize various ligands, including polysaccharides, proteins, lipids, and inorganic compounds [11]. Transmembrane CTLs anchor to the cell membrane, transmitting extracellular signals to intracellular molecules or mediating cell adhesion through interactions between surface proteins [27]. Homologous and phylogenetic analyses revealed that LvLL shares the highest similarity (42.86%) with ladderlectin from *S*. *salar*, *S*. *trutta*, and *O*. *kisutch*, suggesting low conservation of ladderlectin during evolution.

To further verify the function of *LvLL* in shrimp infected with WSSV, RT-PCR was used to analyze the expression levels of *LvLL* in the hepatopancreas, gill, and muscle of healthy and WSSV-infected *L*. *vannamei*. The results showed that *LvLL* was primarily expressed in the hepatopancreas of *L*. *vannamei*. Most reported shrimp CTLs exhibit tissue-specific expression at the mRNA level. For example, *LvLT* [28], *PmLT* [29], and *PmAV* [30] in *L*. *vannamei* and *Fc-hsL* [31] in *P*. *chinensis* were only expressed in the hepatopancreas. *Fclectin* [32] and *PmLec* [33] in *P*. *chinensis* were exclusively expressed in hemocytes. The inducible expression profiles of CTLs upon immune challenge strongly suggest their involvement in host–pathogen interactions [34]. After the WSSV challenge, the expression levels of *LvLL* in the hepatopancreas, gill, and muscle varied significantly, indicating that *LvLL* might be involved in the immune response to pathogen infection. This change was consistent with the expression changes of many CTLs in shrimp upon pathogen infection, such as *LvLectin-1*, *LvLectin-2* [35], and *LvCTL1* [21] from *L*. *vannamei*; FcLec3 [36] and FcLec5 [37] from *P*. *chinensis*; and FmLC5 [38] from *F*. *merguiensis*.

In this study, the knockdown of *LvLL* in *L*. *vannamei* significantly reduced WSSV viral load and increased shrimp survival, suggesting that *LvLL* may promote WSSV infection. Consistent with the results of this study, knocking down *LvLdlrCTL* in *L*. *vannamei* resulted in a significant decrease in the viral load of WSSV, and the mortality rate of shrimp infected with WSSV was significantly reduced [39]. Research on the function of *L*. *vannamei LvCTL 4.2* revealed that knocking down *LvCTL 4.2* led to the inhibition of WSSV replication, implicating that *LvCTL 4.2* may be associated with WSSV infection [23]. The transmembrane PcTlec in *P*. *clarkii* interacted with the VP28 of WSSV, enhancing infection [22]. In mammals and insects, some CTLs also facilitate viral infections. The CTL LSECtin, expressed in human liver and lymph node sinusoidal endothelial cells, was glycosylated at the N-terminus and interacts with glycoproteins of filoviruses and the S protein of severe acute respiratory syndrome coronavirus (SARS), enhancing viral infection [40]. The CTL receptor DCIR on dendritic cells promoted infection by interacting with human immunodeficiency virus type 1 (HIV-1) [41].

To further explore the reasons for enhanced immunity in *L*. *vannamei* following *LvLL* knockdown, we analyzed the expression levels of immune-related genes, focusing on those involved in apoptosis and the proPO activation system. Bcl-2 was essential in the mitochondrial apoptotic pathway [42]. Research has indicated that upon host stimulation, *Bcl-2* inhibits apoptosis, extending the lifespan of infected host cells, facilitating viral replication, and leading to latent and persistent infections [43,44]. The downregulation of *Bcl-2* expression levels and the reduction in the WSSV viral load in this study may indicate that *Bcl-2* plays a role in inhibiting host cell infection by WSSV (Figure 7A). Caspase-8, a proapoptotic protease, is essential in lymphocyte activation and protective immunity [45]. The p53 protein can act directly or indirectly at multiple levels in apoptosis regulation through the induction of numerous apoptotic target genes and transcription-independent mechanisms [46,47]. In this study, the significant downregulation of the apoptosis-related gene caspase 8 (Figure 7B), along with the significant upregulation of *p53* after knocking down *LvLL* (Figure 7C), indicates that apoptosis-related genes play an important role in WSSV infection. Additionally, the expression level of *proPO* was significantly upregulated in this study (Figure 7D). The melanization cascade, activated by the proPO system, has been documented as an important immune mechanism in shrimp and other arthropods for defending against pathogens [48,49]. In *Eriocheir sinensis*, the antibacterial PRR EsLecB participated in innate immune responses by stimulating the proPO activation system to clear pathogens [50].

Deviation from the HWE in a population indicates possible inbreeding, population stratification, or individuals being affected by specific diseases or different selective pressures [51]. Such deviations can also provide evidence for associations in populations in which individuals may be under particular selective pressures or affected by specific ailments [52]. In this study, although the three selected SNPs showed no significant association with WSSV resistance traits, SNP1 deviated from the HWE in both susceptible and resistant groups (Table 2). This suggests possible selective pressure or population structure issues.

## 5. Conclusions

Overall, *LvLL* cloned from *L*. *vannamei* was primarily expressed in the hepatopancreas. The post-WSSV infection expression levels of *LvLL* in the hepatopancreas, gill, and muscle of shrimp significantly changed. Specifically, *LvLL* expression in the hepatopancreas was upregulated 11.35-fold at 228 h after WSSV infection. When *LvLL* was knocked down, the viral load of WSSV in infected shrimp was significantly reduced, and the survival rate of the shrimp increased significantly. Additionally, knocking down *LvLL* resulted in a significant downregulation of the apoptosis-related genes *Bcl-2* and caspase 8, a significant upregulation of *p53*, and a significant upregulation of *proPO* in the WSSV-infected shrimp. In summary, *LvLL* is an important receptor gene that promotes WSSV replication and affects the survival rate of *L*. *vannamei*. Furthermore, further research is needed to elucidate the specific molecular mechanisms of *LvLL* in the interaction between WSSV and *L*. *vannamei*.

## Figures and Tables

**Table 1:** The sequences of primers and probes used in this study.

Primer or Probe Name	Sequences (5′–3′)
LvLL-F	CTTGAAGGCAACACAAACGC
LvLL-R	TTTGCATTCCTCAACTAAAACTACA
qLvLL-F	TTCGTGCTCCTGCTGTCCTT
qLvLL-R	CAAACTCTCTGGGCGTTGGGT
18S-F	TATACGCTAGTGGAGCTGGAA
18S-R	GGGGAGGTAGTGACGAAAAAT
WSSV-F	TGGTCCCGTCCTCATCTCAG
WSSV-R	GCTGCCTTGCCGGAAATTA
WSSV-probe	AGCCATGAAGAATGCCGTCTATCACACA
Bcl-2-F	GCTATGTGTCCTTTGTGGCT
Bcl-2-R	TGAACTTGGCAATGGTAACTG
p53-F	CCCCACATCCACGGAGATA
p53-R	CAATGGCGGCTGATACACC
caspase 8-F	GGCGACAAGATGAGGCAA
caspase 8-R	CAGGGTGAGGGAGAGAAAACT
proPO-F	TACATGCACCAGCAAATTATCG
proPO-R	AGTTTGGGGAAGTAGCCGTC
dsGFP-Fi	GCGTAATACGACTCACTATAGGTGGTCCCAATTCTCGTGGAAC
dsGFP-Ri	GCGTAATACGACTCACTATAGGCTTGAAGTTGACCTTGATGCC
dsLvLL-Fi	GCGTAATACGACTCACTATAGGCGCGCAAAATGATGTTCTTCGT
dsLvLL-Ri	GCGTAATACGACTCACTATAGGTGGAGCGCTACAATCATAATCAAA

**Table 2:** Analysis of SNP mutation types and polymorphism in the *LvLL* gene between S and R groups.

Name	Location (bp)	Type of Base	*H*_o_		*H*_e_		MAF		PIC		HWE (*p*-Value)	
			S Group	R Group	S Group	R Group	S Group	R Group	S Group	R Group	S Group	R Group
SNP1	ORF-23	T/C	0.200	0.275	0.455	0.492	0.350	0.438	0.351	0.371	0.000	0.009
SNP2	ORF-204	C/T	0.450	0.600	0.455	0.495	0.350	0.450	0.351	0.372	1.000	0.335
SNP3	ORF-492	T/A	0.025	0.000	0.025	0.000	0.013	0.000	0.024	0.000	1.000	1.000

**Table 3:** Correlation analysis of SNPs and WSSV resistance traits in the *LvLL* gene.

Name	Genotype	S Group	R Group	χ^2^	*p*-Value
SNP1	TT	22	12	5.230	0.073
	CC	10	17		
	CT	8	11		
SNP2	TT	17	10	2.763	0.251
	CC	5	6		
	CT	18	24		
SNP3	TT	39	40	1.013	0.314
	AT	1	0		

## Data Availability

The original contributions presented in the study are included in the article; further inquiries can be directed to the corresponding author/s.
